# Obeticholic Acid Induces Hepatoxicity Via FXR in the NAFLD Mice

**DOI:** 10.3389/fphar.2022.880508

**Published:** 2022-05-09

**Authors:** Chuangzhen Lin, Bingqing Yu, Lixin Chen, Zhaohui Zhang, Weixiang Ye, Hui Zhong, Wenke Bai, Yuping Yang, Biao Nie

**Affiliations:** ^1^ Department of Gastroenterology, The First Affiliated Hospital of Jinan University, Jinan University, Guangzhou, China; ^2^ Department of Gastrointestinal Endoscopy of Dongpu Branch, The First Affiliated Hospital of Jinan University, Jinan University, Guangzhou, China; ^3^ Department of Gastroenterology, Nanfang Hospital, Southern Medical University, Guangzhou, China

**Keywords:** nonalcoholic fatty liver disease (NAFLD), obeticholic acid (OCA), farnesoid X receptor (FXR), hepatic fibrosis, interleukin -1β

## Abstract

**Objective:** Obeticholic acid (OCA), a potent farnesoid X receptor (FXR) agonist, is a promising drug for nonalcoholic fatty liver disease (NAFLD); however, it can cause liver injury, especially at high doses. Here, we investigated the role of FXR in the high-dose OCA-induced hepatoxicity in the condition of the NAFLD mouse model.

**Methods:** Wild-type (WT) mice and FXR^−/−^ mice were administered with over-dose OCA (0.40%) and high-dose OCA (0.16%), in a high-fat diet. RNA-seq on liver samples of mice fed with high-dose OCA was performed to dig out the prominent biological events contributing to hepatic fibrosis.

**Results:** Over-dose OCA induced liver injury and shortened survival in WT mice, but not FXR^−/−^ mice. High-dose OCA caused hepatic stellate cell activation and liver fibrosis in the presence of FXR. Furthermore, high-dose OCA induced cholesterol accumulation in livers via the upregulation of genes involved in cholesterol acquisition and downregulation of genes regulating cholesterol degradation in liver, leading to the production of interleukin -1β and an FXR-mediated inflammatory response.

**Conclusion:** The high-dose OCA induced FXR-dependent hepatic injury via cholesterol accumulation and interleukin -1β pathway in the NAFLD mice.

## Introduction

Nonalcoholic fatty liver disease (NAFLD) is the most common chronic liver disease worldwide (Fan et al., 2017; Younossi et al., 2018), and becoming a major cause of liver transplantation (Eslam and George, 2020). However, there are no medical treatment available for NAFLD. Farnesoid X receptor (FXR) is a novel therapeutic target for liver diseases. Activation of FXR suppresses hepatic *de novo* fatty acid synthesis (Watanabe et al., 2004), negatively regulates inflammatory response (Wang et al., 2008; Mencarelli et al., 2009; Armstrong and Guo, 2017; Hao et al., 2017), and protects against cholestatic liver damage (Liu et al., 2003). Obeticholic acid (OCA), a potent and selective FXR agonist (Pellicciari et al., 2002), is a Food and Drug Administration-approved therapy for primary biliary cholangitis and is a promising drug for NAFLD (Chapman and Lynch, 2020). Nonetheless, it has been reported that OCA resulted in side effects, including pro-atherogenic lipoprotein profile changes (Neuschwander-Tetri et al., 2015), pruritus (Neuschwander-Tetri et al., 2015; Younossi et al., 2019), liver injury especially in patients exposed to high-dose OCA(Chapman and Lynch, 2020). Recently, the U.S. Food and Drug Administration restricted the use of OCA in patients with primary biliary cholangitis and advanced cirrhosis because it may cause liver failure, which sometimes requires liver transplantation (Eaton et al., 2020). Plasma OCA concentrations were markedly elevated (13-fold) in patients with severe hepatic impairment compared with healthy volunteers (Edwards et al., 2016). Thus, OCA caused deterioration of hepatic decompensation in patients with advanced cirrhosis might due to high concentration of OCA. However, the mechanisms underlying high-dose OCA-induced hepatoxicity and the role of FXR in the high-dose OCA-induced hepatic injury are unclear. The purpose of this study was to explore the mechanisms of high-dose OCA-induced hepatoxicity in the context of FXR activity.

## Materials and Methods

### Animal Experiments

All animal experiments were approved by the Laboratory Animal Ethics Committee of Jinan University. Wild-type (WT) mice and whole body FXR knock out (FXR^−/-^) mice were purchased from Cyagen Biosciences (China). All mice were on a C57BL/6 background. Animals were maintained under specific pathogen-free conditions and had free access to water and food. Eight-week-old male mice were fed a high-fat diet (HFD; 60% kcal from fat) mixed with different doses of OCA (0.04%, normal-dose; 0.16%, high-dose; 0.40%, over-dose; HFD + OCA group) or without OCA (HFD group). Body weight and food consumption were recorded weekly.

### Serum Biochemical Analysis and Hepatic Cholesterol Content Measurements

Serum total cholesterol, alanine aminotransferase (ALT), and aspartate aminotransferase (AST), were determined using enzymatic methods kits (Nanjing Jiancheng, China). To measure liver total cholesterol content, 0.1 g of each liver was lysed and measured by a cholesterol kit (Nanjing Jiancheng, China) according to the manufacturer’s protocol.

### Hepatic Hydroxyproline Content

Liver tissue (70 mg) was hydrolyzed in HCl (6N) as previously described (Trebicka et al., 2007). Hepatic hydroxyproline levels were determined using a hydroxyproline assay kit (Nanjing Jiancheng, China) in accordance with the manufacturer’s protocol.

### Western Blots

An equal amount of denatured protein was separated by SDS-PAGE gel and transferred to a PVDF membrane (Millipore, United States). The membranes were blocked and incubated with anti-IL-1β (abcam, ab9722), anti-NLRP3 (ABclonal, A12694) or anti-GAPDH (Proteintech, 60,004-1-lg) overnight. After an incubation with horseradish peroxidase-conjugated antibodies, protein bands were visualized using chemiluminescence kit (Millipore, United States) and LAS Chemiluminescent Imaging System (LAS500, United States). The bands were quantified by ImageJ.

### RNA Sequencing (RNA-Seq) Analysis

Total RNA from liver was extracted for complementary DNA (cDNA) library construction. Single-end libraries were sequenced by BGISEQ-500, and Bowtie2 software (version 2.2.5) was used to align clean reads to mouse genes and genomes. Gene expression levels (fragments per kilo base of exon per million fragments mapped) were quantified by RSEM (version 1.2.8). Read counts were inputted to DESeq2 to calculate differential gene expression and statistical significance. Differentially expressed genes (DEGs) (HFD + OCA group vs HFD group) were identified as 1) a fold change larger than 2, and 2) *Q*-value (adjusted *p*-value) less than 0.001. Pathways overrepresented by DEGs were annotated in the Kyoto Encyclopedia of Genes and Genomes (KEGG) database. Pathways with a *Q*-value (corrected *p*-value) less than 0.05 was considered significantly enriched. Gene Set Enrichment Analysis (GSEA) software (version 4.1) was utilized to identify enriched signaling pathways. Annotated gene sets of gene ontology (GO) biological processes were used as enrichment inputs. The mouse version of MSigDB gene sets were obtained from Walter and Eliza Hall Institute website (https://bioinf.wehi.edu.au/MSigDB/v7.1/). Enriched pathways were identified as a *p*-value < 0.05 and a false discovery rate (FDR) *Q*-value < 0.25.

### Real-Time Quantitative PCR

Total RNA was extracted from liver using TRIzol (Thermo Fisher, United States) and reverse transcribed using a reverse transcriptase kit (TAKARA, China). The cDNA and SYBR GREEN Premix EXtaq (TAKARA, China) were used for real-time quantitative PCR in a CFX96 Real-Time PCR Detection System. Primer sequences are listed in Supplementary Table S1.

### Statistical Analysis

Data are displayed as mean ± standard error. Comparisons between two groups were evaluated by a two-tailed unpaired Student’s t-test. *p* < 0.05 was considered statistically significant.

## Results

### Over-Dose OCA Caused Hepatic Injury and Impaired Mice Survival in an FXR-dependent Manner

**FIGURE 1:**
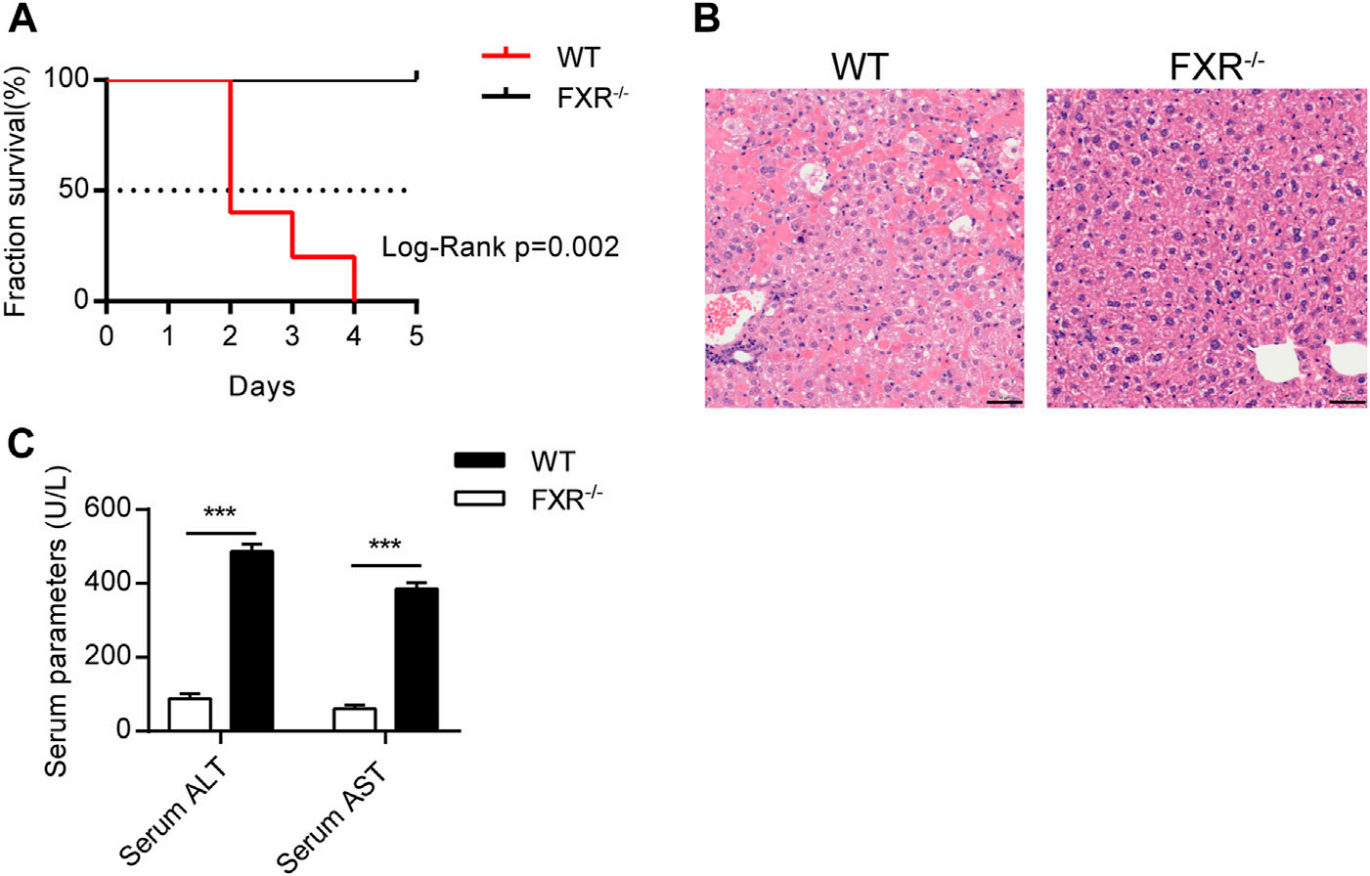
Over-dose OCA reduced survival and promoted hepatic injury in an FXR-dependent manner. Wild-type (WT) and FXR^−/−^ mice were given a high dose of OCA (0.40% OCA incorporated into a high-fat diet) for 5 days (n = 5). **(A)** Survival of WT and FXR^−/−^ mice; Log-Rank *p* = 0.002; HR = 26.85, 95% CI (3.50, 205.70). **(B)** HE staining of liver sections from WT mice and FXR^−/−^ mice. **(C)** Serum alanine aminotransferase (ALT) and serum aspartate aminotransferase (AST) of WT mice and FXR^−/−^ mice. ns, not significant; *, *p*＜0.05; **, *p*＜0.01; ***, *p*＜0.001.

To explore whether FXR is involved in OCA-induced hepatoxicity, wild-type (WT) mice and FXR^−/-^ mice were administered with an over-dose OCA (0.40% incorporated into an HFD) for 5 days. Mice were humanely euthanatized when they were unable to ambulate. During the 5-days period, over-dose OCA caused all of WT mice died, but none of FXR^−/-^ mice died (Figure 1A). Liver slices of WT mice showed disordered and necrotic hepatocytes, as well as infiltrated inflammatory cells (Figure 1B). Furthermore, compared to the FXR^−/-^ mice, WT mice displayed a marked elevation in both serum ALT and serum AST on an over-dose OCA (Figure 1C).

### High-Dose OCA Induced Hepatic Stellate Cell Activation and Liver Fibrosis in the Presence of FXR

**FIGURE 2:**
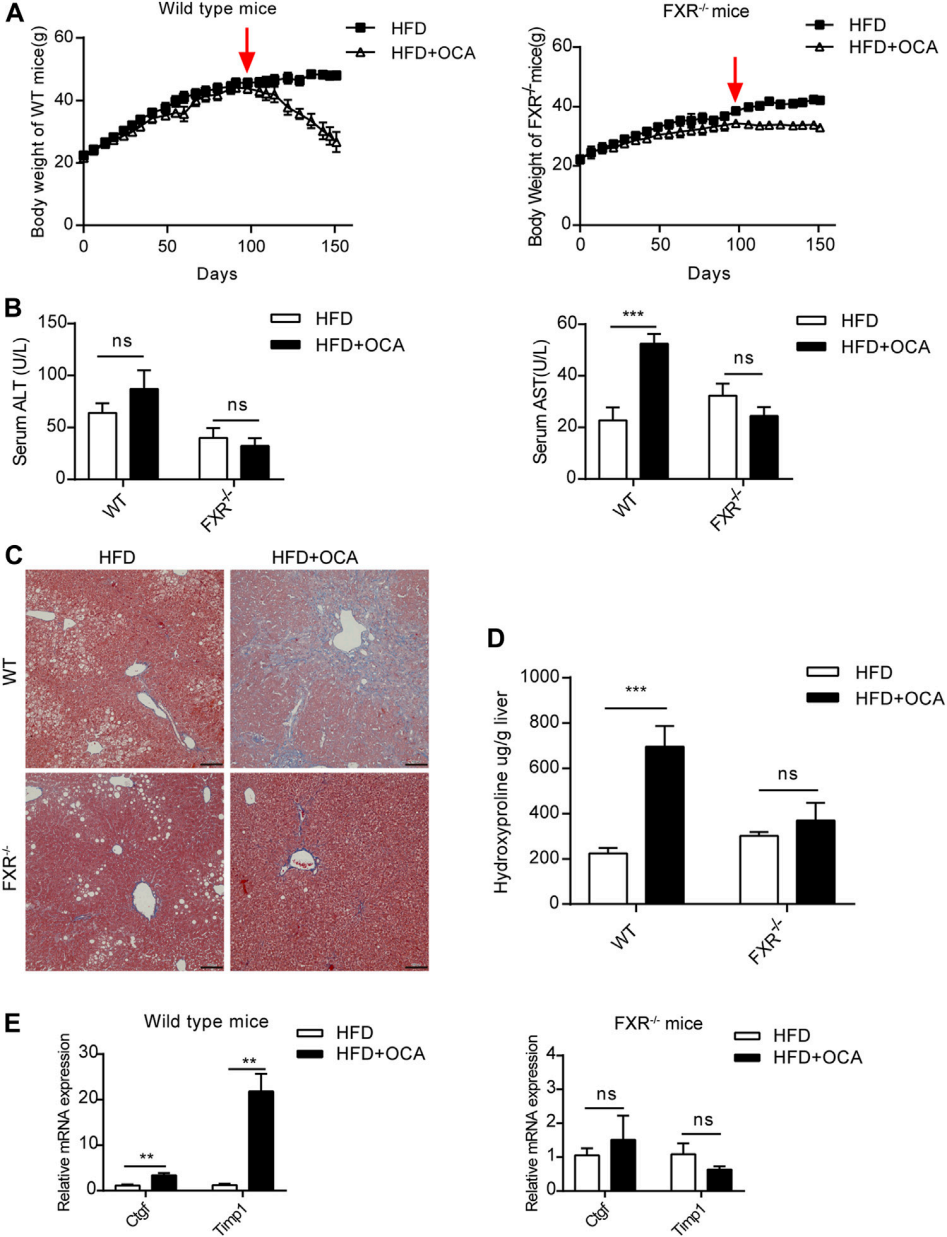
High-dose OCA caused hepatic fibrosis and hepatic stellate cell activation in the presence of FXR. Mice were administered with a high-fat diet supplemented with 0.04% OCA for 14 weeks, after which mice received a high-fat diet containing a high dose of OCA (0.16%, red arrows) for 8 weeks (*n* = 3–6). **(A)** Left panel, body weight of WT mice; right panel, body weight of FXR^−/−^ mice. **(B)** Serum parameters of WT mice and FXR^−/−^ mice. **(C)** Masson’s staining of liver sections from WT mice and FXR^−/−^ mice. **(D)** Hepatic hydroxyproline content. **(E)** Ctgf and Timp1 was determined by quantitative real-time PCR in WT mice and FXR^−/−^ mice. ALT, alanine aminotransferase; AST, aspartate aminotransferase; FXR, farnesoid X receptor; OCA, obeticholic acid; WT, wild-type; ns, not significant; *, *p*＜0.05; **, *p*＜0.01; ***, *p*＜0.001.

Over-dose OCA (0.4% OCA) is lethal, therefore, a non-lethal but toxic dose was used for exploring the mechanisms leading to hepatoxicity. WT mice and FXR^−/-^ mice were fed with 0.04% (normal dose) OCA for 14 weeks, after which the dose of OCA was increased to 0.16% (high dose) for 8 weeks. During the 22-week period, none of the mice died. However, the body weight of WT mice dropped sharply after the dosage increase (Figure 2A, left panel), while the decrease in body weight of FXR^−/-^ mice was mild (Figure 2A, right panel). Furthermore, high-dose OCA led to increased serum AST levels (Figure 2B, right panel) in WT mice, which are indicative of liver injury. The high-dose OCA induced hepatic fibrosis in WT mice but not in FXR^−/-^ mice, as shown by Masson’s staining liver slices (Figure 2C) and increased hepatic hydroxyproline content (Figure 2D). The mRNA expression of tissue inhibitor of matrix metalloproteinase 1 (*Timp1*) and connective tissue growth factor (*Ctgf*), markers of hepatic stellate cell activation, were markedly upregulated in the livers of WT mice (Figure 2E, left panel). FXR deficiency abolished the effect of high-dose OCA on hepatic stellate cell activation (Supplementary Figure S1A). Hence, FXR was essential for high-dose OCA-induced hepatic stellate cell activation and liver fibrosis. Furthermore, high-dose OCA decreased the hepatic TG content in WT mice and FXR^−/-^ mice (Supplementary Figures S2A,B).

### High-Dose OCA Caused an FXR-Mediated Inflammatory Response

**FIGURE 3:**
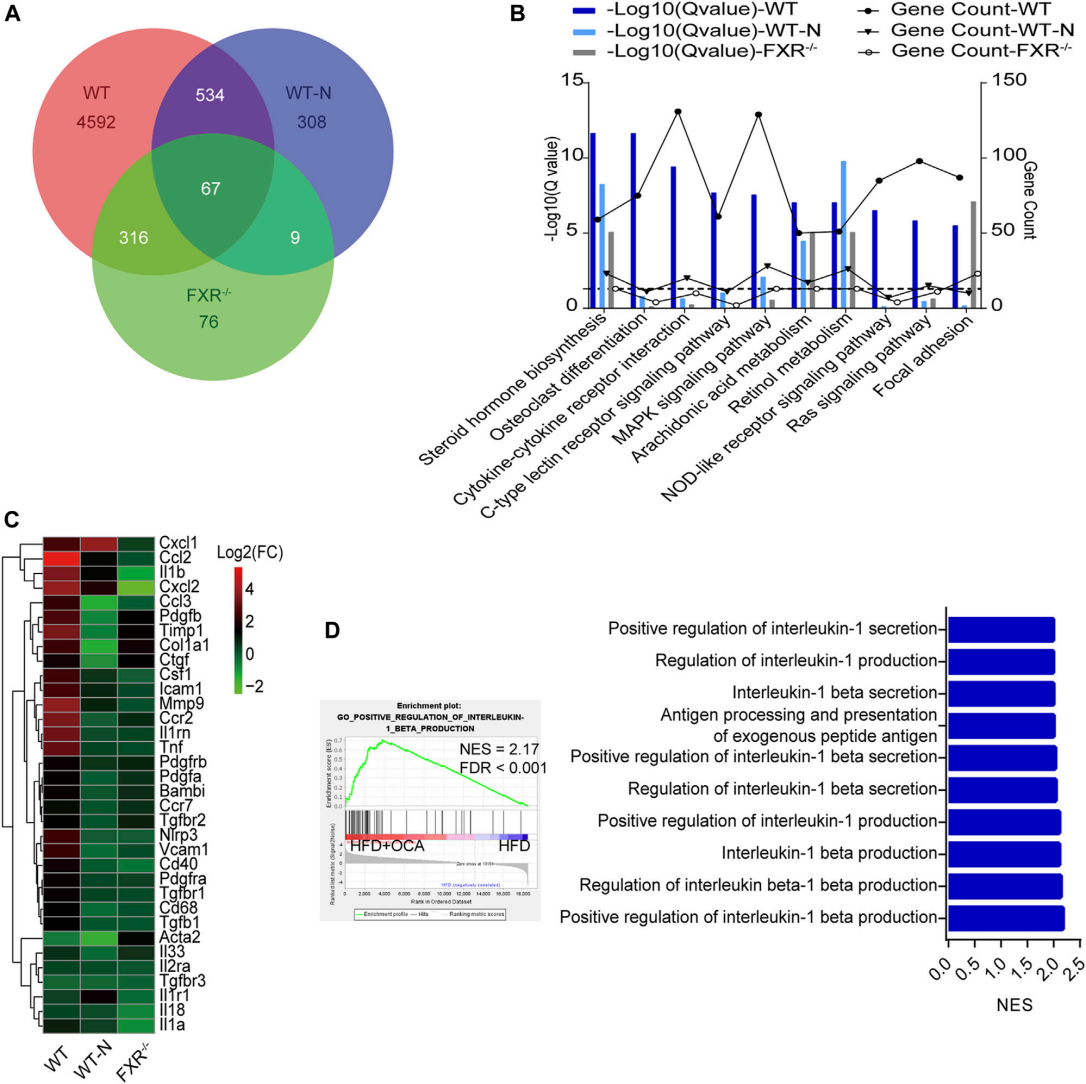
High-dose OCA induced an FXR-mediated inflammatory response WT mice (WT group), and FXR^−/-^ mice (FXR^−/-^ group) fed with 0.16% OCA as described in Figure 2; and WT mice treated with 0.04% OCA for 76 days (WT-N group). **(A)** Differentially expressed genes of mice. **(B)** Kyoto Encyclopedia of Genes and Genomes (KEGG) pathway analysis of differentially expressed genes, dashed horizontal line is plotted at 1.3 (*Q*-value = 0.05). **(C)** Proinflammatory mediator and fibrosis gene expression in livers of mice. **(D)** Gene Set Enrichment Analysis was performed to analyze the pathways enrichment in WT group. Left panel, the most significantly enriched gene sets. Right panel, representative 10 significantly enriched gene sets from GSEA analysis. ES, enrichment score; FDR, false discovery rate; FXR, farnesoid X receptor; OCA, obeticholic acid; NES, normalized enrichment score; ns, not significant; *, *p*＜0.05; **, *p*＜0.01; ***, *p*＜0.001.

**FIGURE 4:**
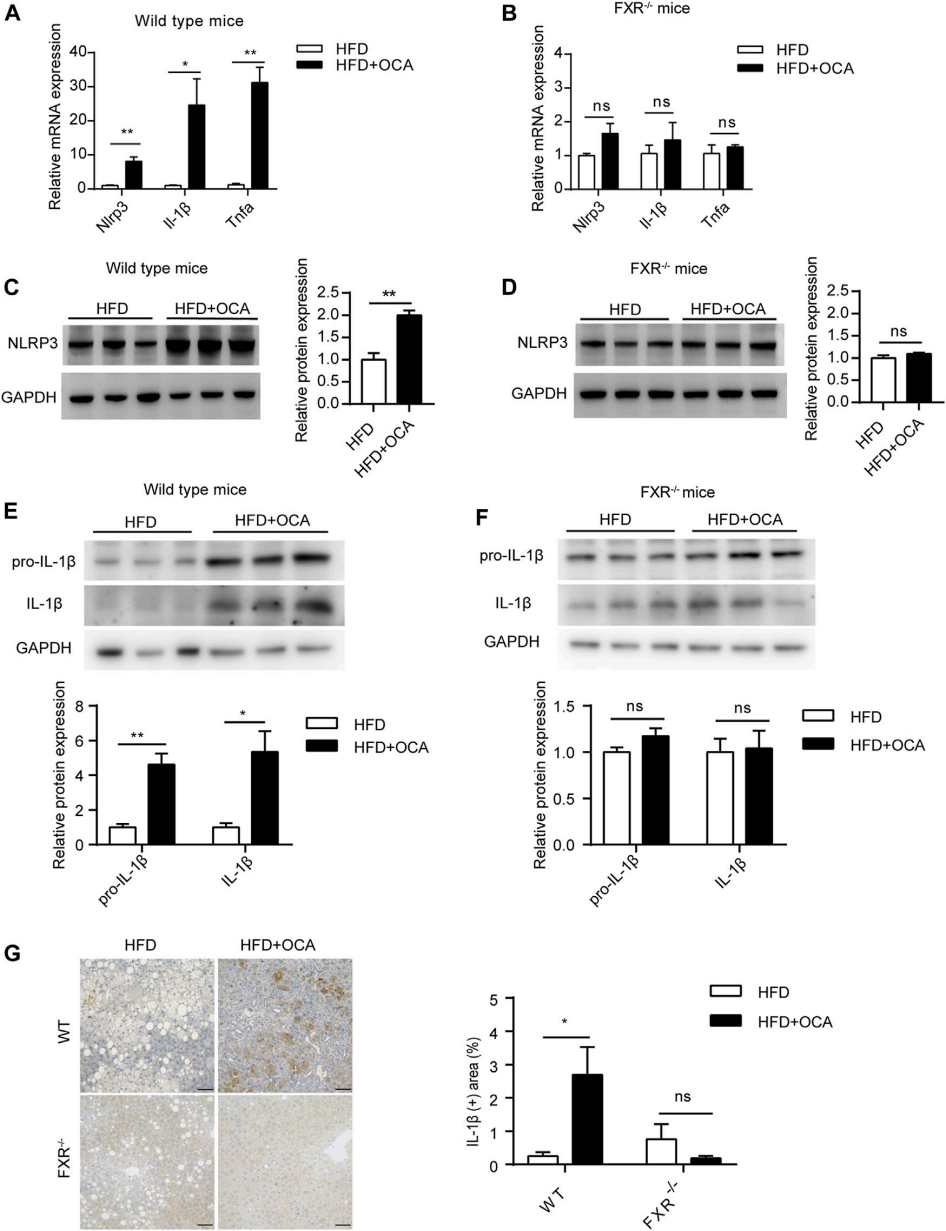
High-dose OCA upregulated IL-1β dependent of FXR. **(A)** Relative mRNA expression of Nlrp3, Il-1β, and Tnfα was determined by quantitative real-time PCR (qPCR) in wild type mice. **(B)** Relative mRNA expression was determined by qPCR in FXR^−/−^ mice. **(C)** Protein expression of NLRP3 in WT group. **(D)** Protein expression of NLRP3 in FXR^−/−^ mice. **(E)** Protein expression of IL-1β in WT group. **(F)** Protein expression of IL-1β in FXR^−/−^ mice. **(G)** Immunohistochemistry for IL-1β in the liver of the mice. (Scale bars, 50 μm). OCA, obeticholic acid; ns, not significant; *, *p*＜0.05; **, *p*＜0.01; ***, *p*＜0.001.

To explore the mechanism underlying high-dose OCA-induced hepatic fibrosis, RNA-seq was performed on the livers of WT mice (WT group) and FXR^−/-^ mice (FXR^−/-^ group) fed high-dose OCA, and WT mice fed normal-dose OCA (WT-N group). A total of 5,509 DEGs were found in WT group, while 468 DEGs in FXR^−/-^ group and 918 DEGs in WT-N group (Figure 3A). KEGG enrichment analysis of DEGs highlighted six enriched pathways in WT group (compared to FXR^−/-^ group) that were related to 1) inflammasome activation and inflammatory responses (cytokine-cytokine receptor interaction, NOD-like receptor signaling pathway, c-type lectin receptor signaling pathway), and 2) fibrogenic responses (osteoclast differentiation, MAPK signaling pathway, and Ras signaling pathway) (Figure 3B). The enrichment of these pathways was almost consistent in the WT-N group and the FXR^−/-^ group (Figure 3B). Furthermore, high-dose OCA upregulated proinflammatory mediators and fibrosis gene expression in livers of WT mice, but not in FXR^−/-^ mice (Figure 3C). However, normal-dose OCA did not induce the expression of these genes in WT mice (Figure 3C). The evidence demonstrated that OCA caused an FXR-mediated inflammatory and fibrogenic response in a dose-dependent manner. GSEA analysis of WT group indicated that the most significantly enriched pathway was positive regulation of IL-1β production (Figure 3D, left panel). Notably, the majority (6 out of 10, 60%) of the most significant pathways in WT group were involved in the IL-1β pathway (Figure 3D, right panel). The mRNA and protein expression of IL-1β were upregulated in livers of WT group (Figures 4A,E,G), but not in FXR^−/-^ group (Figures 4B,F,G). In addition, the expression of *Nlrp3* and *Tnfa* also upregulated in the presence of FXR (Figures 4A–D).

### High-Dose OCA Disrupted Liver Cholesterol Metabolism

**FIGURE 5:**
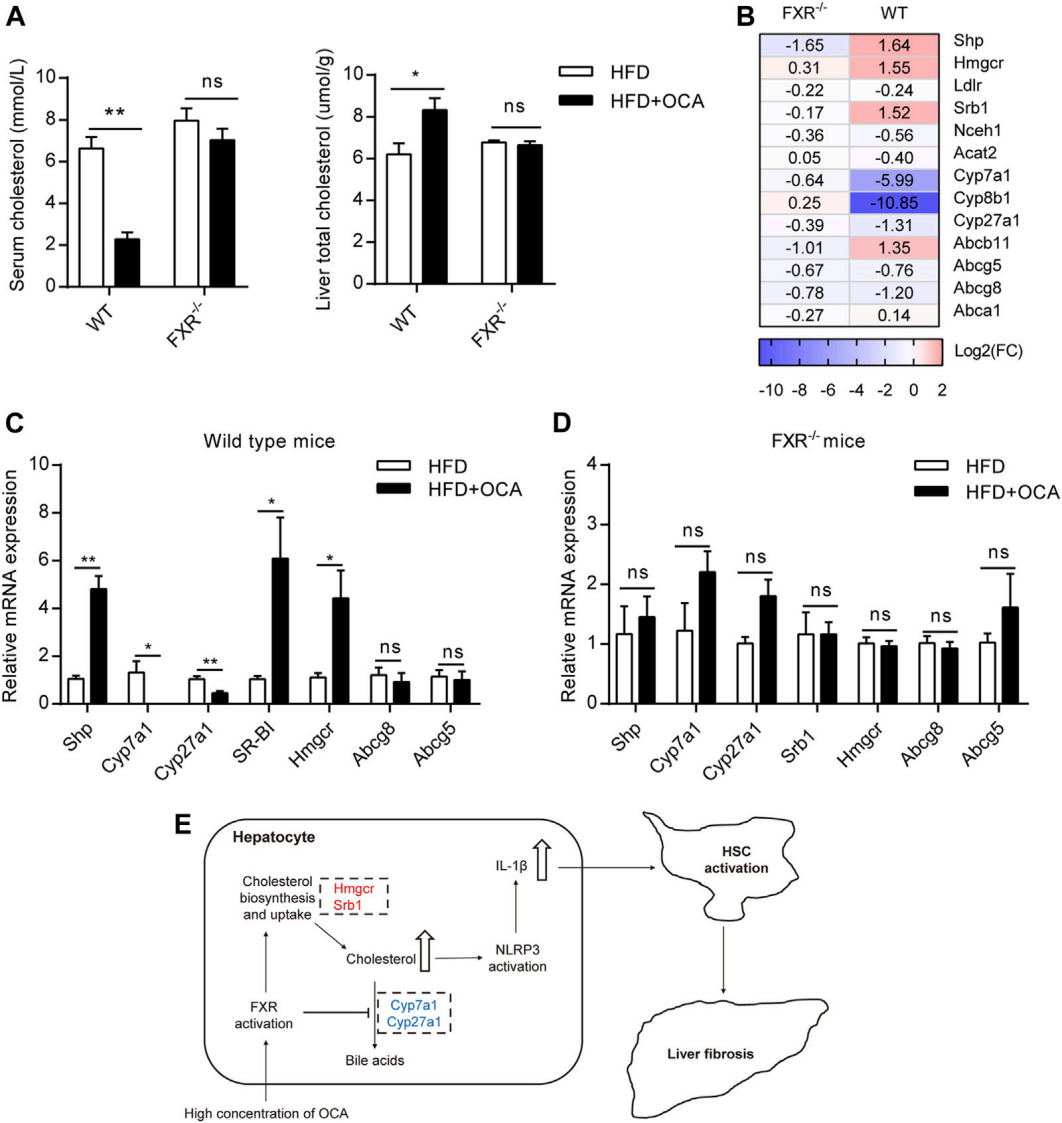
High-dose OCA disrupted liver cholesterol metabolism via FXR activation. Mice were administered with OCA as described in Figure 2. **(A)** Left panel, serum cholesterol; right panel, liver total cholesterol. **(B)** Fold change (FC) of FXR-target genes and cholesterol metabolism genes, as determined by RNA-seq. **(C)** Relative mRNA expression of genes involved in cholesterol metabolism in WT mice, as determined by real-time quantitative PCR (qPCR). **(D)** Relative mRNA expression determined by qPCR in FXR^−/−^ mice. **(E)** Molecular pathways of high-dose OCA-induced hepatic fibrosis (upregulated genes are shown in red; downregulated genes are shown in blue). ns, not significant; *, *p*＜0.05; **, *p*＜0.01; ***, *p*＜0.001.

FXR plays a crucial role in cholesterol homeostasis (Chavez-Talavera et al., 2017). We found that the high-dose OCA decreased serum cholesterol and caused cholesterol accumulation in the livers of WT mice (Figure 5A). The mRNA expression of 3-hydroxy-3-methylglutaryl-CoA reductase (*Hmgcr*), the rate-limiting enzyme for cholesterol synthesis, was upregulated by high-dose OCA in the livers of WT mice (Figures 5B,C). High-dose OCA also upregulated the expression of *Srb1*, which is responsible for hepatic uptake high-density lipoprotein (HDL) cholesterol from circulation. Moreover, high-dose OCA markedly suppressed the expression of enzymes involved in cholesterol conversion to bile acids, such as *Cyp7a1*, *Cyp8b1*, and *Cyp27a1* (Figures 5B,C) in the livers of WT mice. Conversely, the expression of these genes in the livers of FXR^−/-^ mice had no such differences with high-dose OCA treatment (Figures 5B,D). The results revealed that in the presence of FXR, high-dose OCA upregulated genes involved in cholesterol acquisition and downregulated those involved in secretion of cholesterol in the livers. Together, these changes contributed to the cholesterol accumulation in livers. Cholesterol trigger NLRP3 activation, leading to the production of IL-1β, which triggers an inflammatory response, and promotes hepatic stellate cell proliferation (Ioannou, 2016). Therefore, high-dose OCA may have resulted in hepatic fibrosis via cholesterol accumulation and increased the production of IL-1β in an FXR-mediated manner (Figure 5E).

## Discussion

This study revealed that high-dose OCA induced liver injury in an FXR-dependent manner.

OCA was designed as a potent FXR agonist, it alleviates bile acids-induced hepatotoxicity (Trivedi et al., 2016) and NAFLD via FXR activation (Watanabe et al., 2004). However, FXR activation elevated plasma total cholesterol and LDL-c cholesterol levels, and decreased plasma HDL cholesterol levels in human (Neuschwander-Tetri et al., 2015; Pencek et al., 2016; Chavez-Talavera et al., 2017). Thus, FXR activation induces a higher cardiovascular risk lipoprotein profile in human. Interestingly, it is different in mice. FXR activation reduced circulating cholesterol in mice by inhibiting intestinal cholesterol absorption, promoting reverse cholesterol transport in macrophage (Xu et al., 2016), and increasing the expression of hepatic low-density lipoprotein receptor (Singh et al., 2018). In our study, high-dose OCA also reduced serum cholesterol levels in mice.

Even though FXR activation inhibited hepatic cholesterol conversed to bile acid and promoted HDL-c uptakes from circulation (Chavez-Talavera et al., 2017), several studies indicated that a therapeutic concentration of OCA decreased hepatic cholesterol content (Cipriani et al., 2010; Dong et al., 2017; Goto et al., 2018; Morrison et al., 2018). Conversely, in this study, a supratherapeutic concentration of OCA induced hepatic cholesterol accumulation dependent of FXR, which leading to inflammatory response. Previous study shown that activation of FXR suppressed the expression of *Cyp7a1*, *Cyp8b1*, and *Cyp27a1*, and induced the expression of *Srb1* (Li and Chiang, 2014). Importantly, activation of FXR decreased hepatic cholesterol synthesis by suppressing the expression of *Hmgcr* (Hubbert et al., 2007), and promoted cholesterol secretion from hepatocytes by inducing *Abcg8 and Abcg5* expression (Li et al., 2011). These changes caused by FXR activation were responsible for hepatic cholesterol reduction. However, in the present study, high-dose OCA increased the expression of *Hmgcr* without the upregulation of *Abcg8 or Abcg5*. Therefore, high-dose OCA disturbed the hepatic cholesterol homeostasis and induced hepatic cholesterol accumulation.

Cholesterol activated NLRP3 inflammasomes in macrophages contributing to atherogenesis was reported (Duewell et al., 2010; Rajamaki et al., 2010). Accumulating evidence demonstrated that hepatic free cholesterol is a molecular mediator of lipotoxicity (Ioannou, 2016), which activated NLRP3 inflammasomes and upregulated IL-1β, promoting liver inflammation (Mridha et al., 2017). Additionally, blocking NLRP3 activation alleviated hepatic inflammation induced by a methionine/choline-deficient diet and atherogenic diet (Mridha et al., 2017). Hepatic cholesterol accumulation also upregulated expression of TAZ, a profibrotic transcriptional regulator, and leading to liver fibrosis (Wang et al., 2020). Dysregulated cholesterol metabolism results in cholesterol accumulation in the liver of NAFLD patients (Puri et al., 2007; Caballero et al., 2009; Min et al., 2012), which can lead to NLRP3 inflammasome activation (Mridha et al., 2017), hepatic inflammation, and fibrosis (Ioannou, 2016).

Toxicity studies of OCA indicated that the toxicity of OCA to mice fed with a standard diet is primarily on the liver (EUROPEAN MEDICINES AGENCY, 2016). In this study, we explored the mechanism of high-dose OCA-induced hepatotoxicity in the condition of the NAFLD mouse model. The result showed that high-dose OCA resulted in hepatotoxicity in the presence of FXR. The high-dose OCA upregulated the expression of genes involved in the deposition of hepatic cholesterol and decreased the expression of genes involved in cholesterol secretion in livers. This led to an accumulation of cholesterol in liver, which triggered an inflammatory response in the liver, as demonstrated by the increased expression of IL-1β only in the presence of FXR. IL-1β, a potent inflammatory cytokine, promotes hepatic stellate cell proliferation (Reiter et al., 2016) and is involved in the pathogenesis of hepatic fibrosis, the latter of which can be induced by several factors, such as toxins, ethanol, and NASH (Seki and Schwabe, 2015). We demonstrated that FXR plays an important role in OCA-induced liver fibrosis when OCA is administered at high dose or in conditions of advanced cirrhosis, the latter of which resulted in a sharp increase in OCA plasma concentrations (Edwards et al., 2016).

There two limitations of this study. One is that the preclinical toxicology study was performed in mice but not in rats. As we know, the translational value of mice is limited in toxicological studies. Another is that the effect of high-dose OCA on bile acid metabolism was unknown for the measure of hepatic total bile acids was not performed.

In summary, the current study revealed that FXR is essential for high-dose OCA-induced hepatoxicity.

## Data Availability

The datasets presented in this study can be found in online repositories. The names of the repository/repositories and accession number(s) can be found below: https://www.ncbi.nlm.nih.gov/search/all/?term=PRJNA701084.
