# Supplementary figures and images for: Obeticholic Acid Induces Hepatoxicity Via FXR in the NAFLD Mice
Source: Front Pharmacol. 2022 May 9;13:880508. doi: 10.3389/fphar.2022.880508 (PMC9124937; doi:10.3389/fphar.2022.880508)

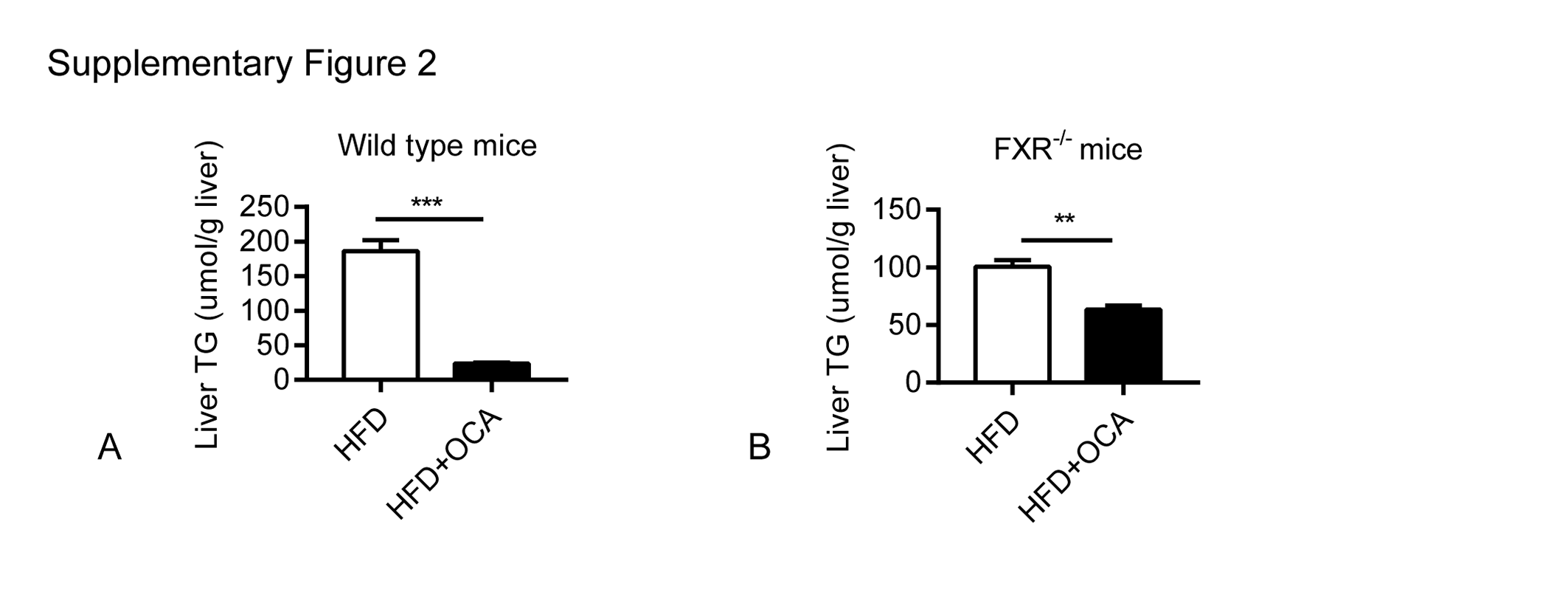

Supplement: Supplementary file 1 [file Image2.TIF]

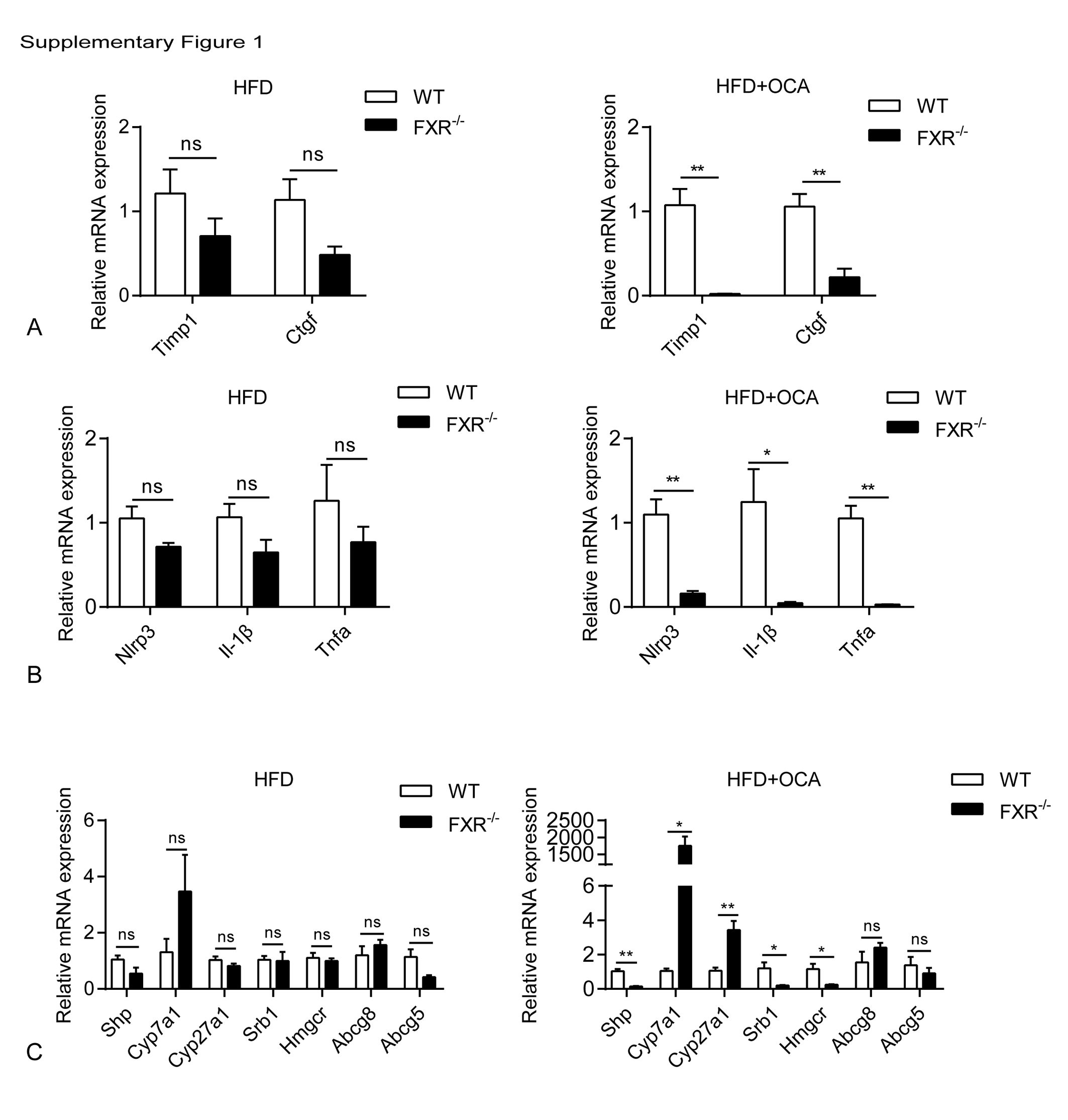

Supplement: Supplementary file 2 [file Image1.TIF]
