# Supplementary material for: Obeticholic Acid Induces Hepatoxicity Via FXR in the NAFLD Mice
Source: Front Pharmacol. 2022 May 9;13:880508. doi: 10.3389/fphar.2022.880508 (PMC9124937; doi:10.3389/fphar.2022.880508)
Supplement: Supplementary file 3 [file DataSheet1.docx]

Supplementary Materials for

**Obeticholic acid induces hepatoxicity via FXR in NAFLD mice**

Chuangzhen Lin^1, †^, Bingqing Yu^1, †^, Lixin Chen^1^, Zhaohui Zhang^1^, Weixiang Ye^2^, Hui Zhong^3^, Wenke Bai^1^, Yuping Yang^1^, Biao Nie^1, *^.

*** Corresponding author. Email: biaonie@jnu.edu.cn**

**Supplementary figure legends**

**Supplementary Figure 1. FXR deficiency altered the effect of high-dose OCA on mRNA expression of gene involved liver fibrosis and cholesterol metabolism.**

(A) Hepatic mRNA expression of *Timp1* and *Ctgf* was determined by quantitative real-time PCR (qPCR) in WT mice and FXR^-/-^ mice. (B) Hepatic mRNA expression of *Nlrp3*, *Il-1β*, and *Tnfα* was determined by qPCR in WT mice and FXR^-/-^ mice. (C) Hepatic mRNA expression of genes involved in cholesterol metabolism in WT mice and FXR^-/-^ mice, as determined by qPCR. ns, not significant; *, P＜0.05; **, P＜0.01; ***, P＜0.001.

**Supplementary Figure 2. High-dose OCA decreased liver TG content in mice**

Liver lipid extracts were prepared by lysing 0.1 g of each liver, and triglyceride (TG) content was determined by a kit. (A)Liver TG content of wildtype mice. (B)Liver TG content of FXR^-/-^ mice. ns, not significant; *, P＜0.05; **, P＜0.01; ***, P＜0.001.

**Supplementary Table 1. Real-time PCR primers.**

| Genes | Sense (5’-3’) | Anti-sense (5’-3’) |
| --- | --- | --- |
| Il-1β | TGGTGTGTGACGTTCCCATT | CAGCACGAGGCTTTTTTGTTG |
| Timp1 | TGGCATCTGGCATCCTCTTG | CGCTGGTATAAGGTGGTCTCG |
| Ctgf | AGAACTGTGTACGGAGCGTG | GTGCACCATCTTTGGCAGTG |
| Nlrp3 | AGCCTTCCAGGATCCTCTTC | CTTGGGCAGCAGTTTCTTTC |
| Tnfα | AGCACAGAAAGCATGATCCG | CTGATGAGAGGGAGGCCATT |
| Srb1 | TGGTCTGAACCCTAACCCAAAG | AGAACTACTGGCTCGATCTTCC |
| Cyp27a1 | GGGTGGACACGACATCCAAC | GCGCAGGGTCTCCTTAATCA |
| Hmgcr | GCTGGTGAGCTGTCCTTGAT | TCCTCAAGCTGCCTTCTTGG |
| Abcg5 | GCCACGGTCATTTTCAGCAG | AGGAATGGGCATTTCTTGTATGT |
| Abcg8 | CCCTGATCCGTCGTCAGATTT | CATTGACCTCTCCGAGTGACAT |
